# Time to recovery and its determinant factors among patients with COVID-19 in Assosa COVID-19 treatment center, Western Ethiopia

**DOI:** 10.1186/s41479-023-00119-3

**Published:** 2023-11-05

**Authors:** Maru Zewdu Kassie, Molalign Gualu Gobena, Yihenew Mitiku Alemu, Awoke Seyoum Tegegne

**Affiliations:** 1 Assosa University, Assosa, Ethiopia; 2 Injibara University, Injibara, Ethiopia; 3 Bahir Dar University, Bahir Dar, Ethiopia

**Keywords:** COVID-19, Time to recovery, Proportional hazard model

## Abstract

**Background:**

The Novel Coronavirus disease (COVID-19) pandemic has become a global threat. Determining the time to recovery from COVID-19 is intended to assist healthcare professionals in providing better care, and planning logistics. So, the study aimed to identify the factors that affect the time to recovery from COVID-19 for patients treated at Assosa COVID-19 treatment center, Benishangul Gumuz Regional State, Western Ethiopia.

**Methods:**

A retrospective study design was conducted on 334 randomly selected COVID-19 patients at Assosa COVID-19 treatment center from February 2021 to July 2021. The median survival time, Kaplan–Meier survival estimate, and Log-Rank test were used to describe the data and compare the survival time between groups. The study used the Cox PH model to analyze the time to the first recovery of COVID-19 patients, where hazard ratio, p-value, and 95% CI for hazard ratio were used for testing significance. Schoenfeld and Cox-Snell residuals were used for checking the model assumption.

**Results:**

The overall incidence rate was 13.79 per 100 (95% CI: 10.04, 18.95) person-days observations. The median time to recovery was 16 days. At the end of the follow-up, 77.2% of the patients had developed an event of recovery, and the rest 22.8% were censored. The mean age of patients was 45.22 years. Severe COVID-19 patients (AHR = 0.7876, 95% CI: 0.7090, 0.8748), presence of symptoms (AHR = 0.2814, 95% CI: 0.1340, 0.5914), comorbidity (AHR = 0.1627, 95% CI: 0.1396, 0.1897), ≥ 90 oxygen saturation (AHR = 3.2370, 95% CI: 2.161, 4.848), and being older age (AHR = 0.9840, 95% CI: 0.971, 0.9973) were found to have statistically significant association with the time to recovery from COVID-19.

**Conclusion:**

The study concludes that severe COVID-19 patients, male patients, patients having comorbidity, older age, and patients having symptoms as poor prognostic factors of COVID-19 disease and also prolonged recovery time. Therefore, health providers in treatment centers should give strict follow-up and priority to older patients, severe COVID-19 patients, and patients having another co-morbid illness by focusing on respiratory difficulties and underlying pre-existing medical conditions to manage the disease severity and recover quickly.

## Introduction

The coronavirus pandemic is an outbreak which is emerged in December 2019 in Wuhan city, China [1]. After three months, the disease was declared a global pandemic by the World Health Organization (WHO) on 11 March 2020 [2, 3]. COVID-19 is an infectious disease caused by a virus called coronavirus. The name Corona represents crown-like spikes on the outer surface of the virus; thus, it was named a coronavirus [4]. Research indicated that COVID-19 is mainly transmitted through respiratory droplets, airborne transmission, fecal–oral transmission, and close contact with another person who has the virus [5, 6].

All age groups are infected by this virus. But, evidence suggests that two groups of people are at a higher risk of getting severe COVID-19 disease. These are older people over 60 years old and who have other related diseases like cardiovascular disease, diabetes, chronic respiratory disease, and cancer are more relatively at risk [7, 8]. Common human coronaviruses including types 229E, NL63, OC43, and HKU1 cause mild to moderate upper respiratory tract [9]. Some of the signs and symptoms include sore throat, runny nose, and cough [10, 11].

As per the WHO daily situation report, the total cases of COVID-19 are increasing worldwide. At the time of writing this manuscript, globally there were more than 555 million confirmed cases and about 6.3 million died out, and 530 million cases were recovered from COVID-19. From these Africa shares 12.3 million cases, 256,000 deaths, and 11.5 million cases recovered. Ethiopia also reported 486,502 cases, 7,542 deaths, and 464,598 cases recovered [12].

Researching factors that affect the time to recovery from COVID-19 is crucially important since disease severity, death, and recovery vary from one individual to another due to different factors. Studies showed that factors of older age and the presence of pre-existing co-morbidities delayed the recovery time from COVID-19 [8, 13–17]. The previous studies found that the median time to recovery from COVID-19 infection of 13 days, with a range of 9–17 days [18]; 18 days with a range of 10–27 days [15]; 11 days with an IQR of 9–14 days [16]; and 19 days with a wide range of 2–71 days [8]. This recovery time variation from one study to another needs further investigation.

The presence of symptoms like respiratory symptoms and other constitutional symptoms was associated with prolonged recovery time from COVID-19 [19–21]. In a study conducted in Zhejiang Province [22], among hospitalized patients with COVID-19; Male patients, immune globulin use, Acute physiological and chronic health Evaluation II (APACHE-II) score, and lymphocyte count were associated with delayed recovery time from COVID-19. Also from research conducted in Italy [23], Body Mass Index (BMI) was associated with prolonged recovery time from COVID-19. A study conducted on the risk factors for delayed viral clearance in COVID-19 infection, in their finding patients having hypertension and intravenous immunoglobulin delay viral clearance in COVID-19 patients [13]. Children under 10 years of age and females had a lower incidence of COVID-19 infection than adolescents or adults and males [24]. Female patients recover in a shorter time as compared to male patients [18, 22]. However, from another study [25] males were significantly more likely to report complete recovery than females (46.1% vs 36.7%, *p* = 0.021). Also from the study [26] about covid-19 situation in India, the recovery time of male and female patients was nearly similar. All these suggest the need for further studies to evaluate the real effects of sex on the time to recovery from COVID-19. All these above studies and other studies in Ethiopia's COVID-19 treatment center [27–29] used the Cox-PH (Cox Proportional Hazard) model without checking the proportional hazard assumption. This assumption is a precondition to using this model. If this assumption is violated the simple Cox model is invalid, and more sophisticated analysis; parametric accelerated failure time models are required [23, 30]. In addition, some of these studies missed including some potential predictors like Oxygen saturation, presence of Asthma, and Disease severity. In our study, we have addressed all these issues by including these missed predictors in our analysis and by checking the assumption using the Schoenfeld residual plot and its standard statistical tests.

At different COVID-19 treatment centers in Ethiopia, there were studies on the time to recover from COVID-19 and its determinant factors [27–29, 31, 32]. Almost all of them provided the same findings and used the same methodology (Cox-PH model). Unfortunately, the finding of one of these studies could not be a generalization for others. This is because they studied in different settings. Conducting several studies in different settings is very crucial to get reasonable and authentic information. Furthermore, it helps to know detailed information about the disease, for example, the pattern of the disease. As a result, health providers can plan and mobilize resources effectively [33, 34]. Still, there is no study on this topic in Benishangul Gumuz Reginal State (BGRS) of Ethiopia particularly in the Assosa COVID-19 treatment center. Therefore, this study aimed to identify the factors that affect the time to recovery from COVID-19 for patients treated at Assosa COVID-19 treatment center, BGRS, Western Ethiopia in different study settings using the Cox-PH model.

## Methods

### Study area and design

The study was conducted at Assosa COVID-19 treatment center, BGRS, Western Ethiopia. Assosa is located 670 km far from Addis Ababa in the Western part of Ethiopia. The treatment center was previously established for routine health center service and later on, was exclusively dedicated to COVID-19 treatment by the regional health bureau. A retrospective study design was carried out from February 2021 to July 2021 at Assosa COVID-19 treatment center to retrieve relevant information from the medical records of patients under quarantine.

### Source of population and data collection procedures

COVID-19 patients who were under follow-up of COVID-19 treatment at Assosa COVID-19 treatment center were the source of the population for this study. The secondary data was collected from the medical chart of COVID-19 patients at the treatment center. The data for this study were collected using a standardized data collection tool specifically designed for COVID-19 patients. The tool included various variables such as demographic information, medical history, laboratory results, treatment regimens, and follow-up visits. Trained healthcare professionals at Assosa COVID-19 treatment center extracted the necessary information from the medical charts of COVID-19 patients who were under follow-up from February 2021 to July 2021.

To ensure accuracy and consistency in data collection, training sessions were conducted for the healthcare professionals involved in extracting data. After the data collection forms were completed, a thorough quality check was conducted to verify that all necessary information was properly collected and recorded. Any missing or inconsistent data were resolved by referring back to the medical charts or contacting the healthcare providers for clarification. Overall, the data collection process was designed to ensure the reliability and validity of the collected data, and efforts were made to minimize any potential biases or errors in the data.

### Inclusion–exclusion criteria

Patients diagnosed with COVID-19 at the Assosa COVID-19 Treatment Center within the study period; Patients who have been admitted to the treatment center for at least 3 days follow-up; Patients who have complete medical records and information available for analysis; and Patients of all age groups were included in this study. While Patients who have not completed their treatment or are still in the early stages of treatment; Patients who have been transferred or discharged from the treatment center before recovery or without complete recovery data; and Patients with missing or incomplete medical records or information necessary for analysis were excluded. These criteria will help ensure that we select a representative sample from our total population while excluding individuals who may introduce bias or confounding factors into our research findings.

Overall, a total of 962 COVID-19 patients were identified during the study period. However, after applying the inclusion–exclusion criteria, only 334 patients met the eligibility criteria and were included in this analysis. These 334 patients were then followed until they either experienced the event of interest or reached censoring. The data were analyzed using the statistical packages SAS version 9.2 and R version 4.00.

### Operational definitions

*Right censoring*: is considered when the patient is not recovered once between the study time, is transferred to another hospital, and died before recovery.

*Time to recovery*: is the time from the start of the treatment until it reaches recovery in the follow-up period.

*COVID-19 Severity*: Categorized as mild, moderate, or severe based on the severity level assigned to each patient's COVID-19 symptoms and clinical condition.

*Mild*: Individuals with mild COVID-19 typically experience mild symptoms similar to a common cold or flu.

*Moderate*: involves more pronounced symptoms that include higher fever, difficulty breathing, headache, chest pain, and gastrointestinal issues like diarrhea or vomiting.

*Severe*: Severe cases of COVID-19 are characterized by severe respiratory distress, such as acute respiratory distress syndrome (ARDS), which can lead to significant breathing difficulties.

### Variables in the study

The outcome variable considered in this study was the time to recover from COVID-19 infection.

$$status = \left\{\begin{array}{ll}0,& if\;censored\\ 1,& if\;event\end{array}\right.$$

Covariates considered were Age in years, Sex (Female, Male), COVID-19 Severity (mild, moderate, severe), Comorbidity (No, Yes), Chronic respiratory disease (No, Yes), Asthma (No, Yes), Presence of symptoms (No, Yes), Oxygen Saturation ($\le 89, \ge 90$) and Respiratory symptoms (No, Yes).

### Survival analysis

Survival Analysis is used to analyze data in which the time until the event is of interest [22]. We used the survival analysis to identify factors that affect the recovery time for covid-19 patients. Descriptive analysis of survival data was presented graphically using the Kaplan–Meier estimator. Log-rank test was used to compare the survival experience of different categories of covariates. The proportional hazard assumption was checked by using the Schoenfeld residual test [23]. Cox proportional hazards (PH) regression model was used to identify the potential risk factors associated with the time to recovery among COVID-19 patients. This model is a semi-parametric model which is based on the assumption of proportional hazards, no particular form of the probability distribution is assumed for the survival times [24]. In this model, the hazard of recovery at time $\mathbf{t}$ can be expressed as:

$$h\left({t}_{,}{x}_{i},\beta \right) = {h}_{0} \left(t\right) \mathrm{exp }\left({x}_{i}^{T}\beta \right)$$

Where, ${h}_{0}(t)$ is the baseline hazard function; ${x}_{i}$ is a vector of covariates and $\beta$ is a vector of parameter estimates. Note that;${h}_{0}(t)$ is the hazard function, where all values of the covariates are zero ($\mathrm{exp}\left({x}_{i}^{T}\beta \right)=1$). Parameter estimate $\beta$ refers to the increase in log-hazard with a one-unit increase for the continuous covariate.

Univariate analysis was performed to calculate an unadjusted hazard ratio (HR) and to screen out potentially significant independent variables at a 25% level of significance. Then multivariate analysis was performed to assess the association between all variables that are significant in the univariate case and the time to recovery from COVID-19 at a 5% level of significance. Adjusted hazard ratio (AHR), *P*-value, and 95% CI (Confidence Interval) were considered to assess whether each independent variable was statistically significant or not. If a variable *P*- value $\le$ 0.05 was considered as statistically associated with the time to recovery from COVID-19 in days.

The maximum likelihood estimation technique was used to estimate the $\beta$ parameters in the proportional hazard model [25]. After fitting the model to the data, the adequacy of the fitted models to the survival data would be checked using Cox-Snell residuals and martingale residuals [26]. A stepwise selection method was performed to select the potential predictor variables that have a strong association with time to recovery from COVID-19 [35].

## Results

### Socio-demographic and clinical variables with their censoring status

The summary statistics of predictor variables in the data were displayed in (Table 1). Of a total of 334 COVID-19 patients, 258(77.2%) got the event of the first recovery and the rest 76(22.8%) were censored. The majority of the patients (51.5%) were males, 47.9% of the patients were unknowing where the diseases hold them, and also 34.7% of the patients have related co-morbid illnesses. The mean and SD (standard deviation) of age at the start of the treatment were 45.22 and 12.15 respectively. The rest variables were described in the same way.

**Table 1:** Summary statistics for independent variables included in the study

Variable	Category	Obs. Event	Censored (%)	Total (%)
Sex	Female	148(44.3)	14(4.2)	162(48.5)
	Male	110(32.9)	62(18.6)	172(51.5)
Severity	Mild	114(34.1)	16(4.8)	130(38.9)
	moderate	132(39.5)	28(8.4)	160(47.9)
	Severe	12(3.6)	32(9.6)	44(13.2)
Co-morbidity	No	150(44.9)	68(20.4)	218(65.3)
	Yes	108(32.3)	8(2.4)	116(34.7)
Respiratory disease	No	216(64.7)	28(8.4)	244(73.1)
	Yes	42(12.6)	48(14.4)	90(26.9)
Presence of asthma	No	190(56.9)	56(16.8)	246(73.7)
	Yes	68(20.4)	20(6.0)	88(26.3)
Presence of symptoms	No	210(62.9)	22(6.6)	232(69.5)
	Yes	48(14.4)	54(16.2)	102(30.5)
Oxygen Saturation	$\le 89$	102(29.7)	4(2.0)	106(31.7)
	$\ge 90$	156(46.8)	72(21.5)	228(68.3)
Respiratory symptom	No	246(73.7)	36(10.8)	282(84.4)
	Yes	12(3.6)	40(12.0)	52(15.6)
Baseline measured continuous covariate				
Age of patient			Mean	SD
			45.22	12.15

Also as indicated in (Table 2) the median recovery time was 16 days.

**Table 2:** Median recovery time

medians for survival time			
Median			
estimate	SE	95% Confidence Interval	
		Lower bound	Upper bound
16.000	0.267	15.477	16.523

### Non-parametric analysis for survival data

#### Kaplan- meier survival curves

**Fig. 1:**
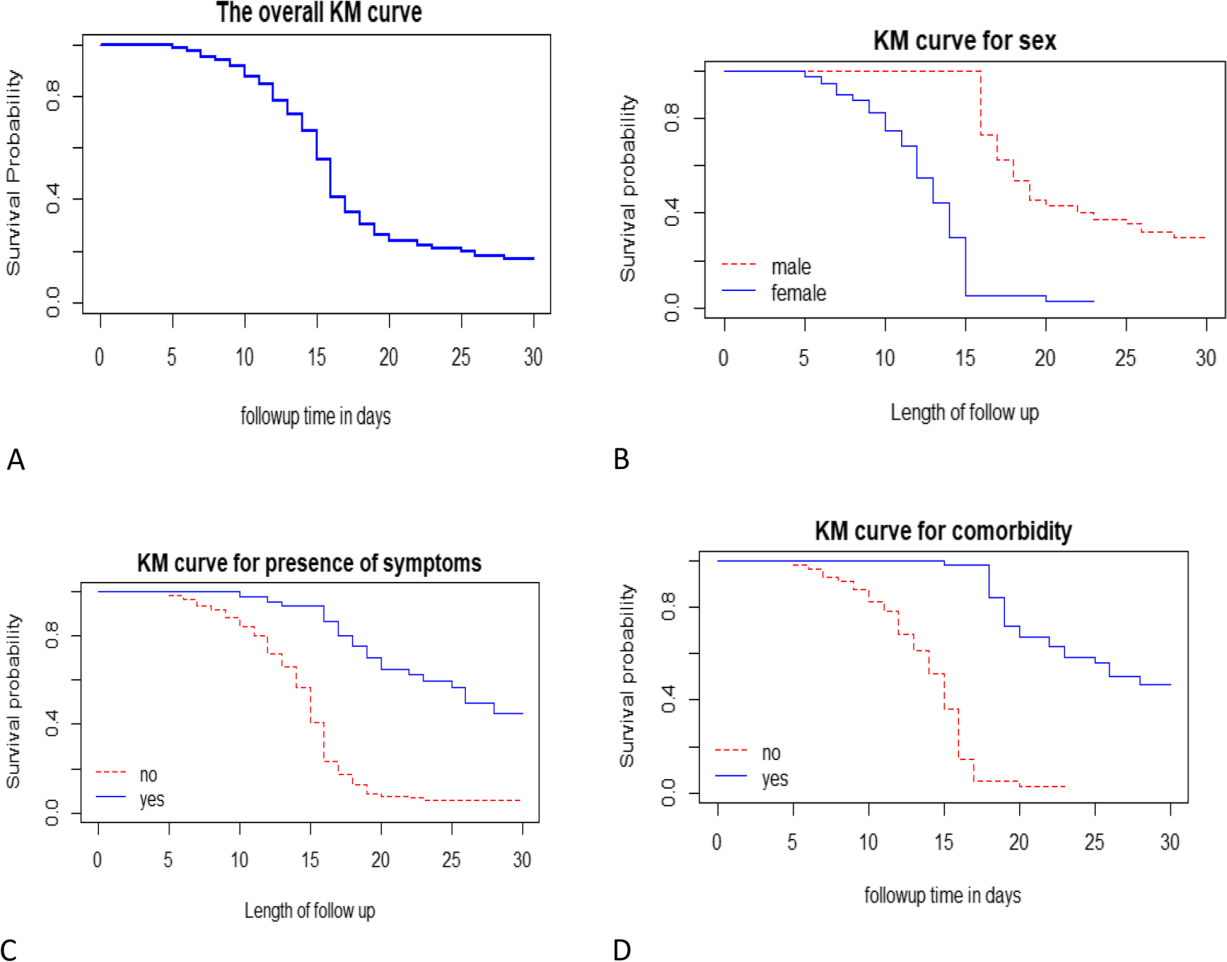
Kaplan- Meier survival estimate curves

In the underneath KM (Kaplan- Meier) plot, recovery time is being measured in person-days. The overall Kaplan–Meier survival curve starts at zero and then the curve drops down until the follow-up recovery time happened at 30 person-days. It indicated that as follow-up time increases the curve decreased rapidly i.e. most patients recovered from COVID-19 as follow-up time increased continuously up to 30 person-days (Fig. 1A).

The plot in (Fig. 1B) suggested that the length of recovery time for male patients was greater than for female patients. That means male patients had taken a long time to recover as compared to female patients. (Fig. 1C) indicates that those COVID-19-positive patients who had one or more COVID-19 symptoms are less likely to recover from COVID-19 than those COVID-19-positive patients who hadn’t symptoms. In contrast, those COVID-19-positive patients who hadn’t related co-morbidity are more likely to recover early from COVID-19 than those who had (Fig. 1D). The remaining covariates could be presented and interpreted in the same way.

### Log-rank test for each categorical variable

Log-rank test was computed to check the significant difference among the categories of categorical variables. The null hypothesis said that there is no significant difference between the survival experiences of different groups of categorical variables. Table 3 displayed the log-rank test of each categorical variable and reveals that there was a significant difference in recovery rate among males and females, having related comorbidity and no related comorbidity, presence, and absence of respiratory disease, having symptoms and not having symptoms, and patients having respiratory symptoms and not having respiratory symptoms. However, there was no difference in recovery rate between the presence and absence of asthma.

**Table 3:** Log-rank tests for categorical variables

Covariates	DF	Chisq	*P*-value
Sex	1	68	< 0.0001
Severity	2	41.4	< 0.0001
Co-morbidity	1	72.91	< 0.0001
Respiratory disease	1	36.4	< 0.0001
Presence of asthma	1	8.9	0.0142
Presence of symptoms	1	62.7	< 0.0001
Respiratory symptoms	1	34.1	< 0.0001

### Cox proportional hazards model

#### Variable selection and Cox PH assumption

The study used a purposeful variable selection method to determine the variables to be included in the survival model. By purposeful variable selection method first test the significances of each predictor variable at a 25% level of significance, then by only the variables significant at this level; we could fit the multivariate Cox proportional hazard model. Consequently, the candidate variables for building a multivariable Cox model were the sex of the patient, age, co-morbidity, presence of respiratory disease, Severity of COVID-19, presence of symptoms, presence of asthma, and presence of respiratory symptoms were candidate variables for multivariable model building.

The proportional hazards assumption asserts that the hazard ratios are constant over time. That means the risk of failure must be the same no matter how long subjects have been followed. To test Cox proportional hazard assumption, a GLOBAL test was used.

From Table 4, the *p*-values of all covariates are greater than 5%, indicating that the correlation between Schoenfeld residuals and survival time is not significant; this implies that all the covariates satisfy the proportionality assumption at a 0.05 level of significance, and also the p-value of the GLOBAL test (0.326) is not significant. This indicates that the PH assumption for the Cox model is not violated.

**Table 4:** Cox proportional hazard assumption test

Variable	Ch-square	Df	*P*-value
Age	1.876	1	0.1708
Sex	3.1068	1	0.0933
Co-morbidity	1.352	1	0.2410
Respiratory disease	0.894	1	0.285
Asthma	0.414	1	0.520
Symptoms	3.217	1	0.0914
Respsymptom	0.406	1	0.517
Oxygen saturation	3.742	1	0.740
Severity	1.463	2	0.287
Global	13.544	10	0.326

### Multivariable analysis for Cox Proportional Hazard (Cox-PH) model

Since the proportional hazard assumption was not violated, the data were analyzed based on Cox proportional hazard model. All of the parameter estimates were estimated by taking the other predictor variables into account using a 95% confidence interval for the hazard ratios of the statistically significant risk factors of COVID-19 which do not include 1 (the null value). In contrast, the 95% confidence intervals for the non-significant risk factors include the null value. Table 5 displayed the result of the multivariable analysis of the Cox proportional hazard model; sex of the patient, presence of co-morbidity, presence of respiratory disease, presence of asthma, presence of symptoms, and presence of respiratory symptoms were significantly associated with time to recovery from COVID-19 at a 5% level of significance.

**Table 5:** The multivariate Cox proportional hazards model analysis

Variable	Category	AHR (95% CI)	*P*-value
Sex	Male	1	
	Female	4.9201(2.1660,11.1718)	0.0001^***^
Age		0.9840 (0.971,0.9973)	0.0181^*^
Disease severity	Mild	1	
	Moderate	1.3796(0.9813,1.9394)	0.0673
	Severe	0.7876(0.7090,0.8748)	< 0.0001^***^
Symptoms	No		
	Yes	0.2814(0.1340,0.5914)	0.0009^***^
Respiratory symptoms	No	1	
	Yes	0.2807(0.1456,0.5412)	0.0001^***^
Presence of Asthma	No		
	Yes	0.5415 (0.3830, 0.7657)	0.0004^***^
Comorbidity	No		
	Yes	0.1627(0.1396,0.1897)	< 0.0001^***^
Oxygen saturation	$\le 89$		
	$\ge 90$	3.2370(2.161, 4.848)	< 0.0001^***^
Respiratory disease	No		
	Yes	0.2301(0.0792,0.6607)	0.0070^***^

^*^Reflect those predictors significant at 5% level of significance

^***^Reflect those predictors significant at both level of significance (i.e at 5% and 1%)

The overall incidence rate was 13.79 per 100 (95% CI: 10.04, 18.95) person-days observations.

According to the result in (Table 5) the rate of achieving recovery for female patients was 4.9251 times higher than for male patients (AHR = 4.9201, 95% CI: 2.1660, 11.1718). That means the time needed to reach recovery for female patients was significantly shorter compared to male patients. The rate of achieving recovery for patients having severe COVID-19 cases was lower by 21.24% compared to patients having mild COVID-19 cases (AHR = 0.7876, 95% CI:0.7090, 0.8748). This means the time needed to reach recovery for patients having severe COVID-19 cases was longer.

Similarly, the rate of achieving recovery for patients who have COVID-19 symptoms was 71.86% times lower than for patients who don’t show COVID-19 symptoms (AHR = 0.2814, 95% CI: 0.1340, 0.5914). That means the time needed to reach recovery for patients who had COVID-19 symptoms take longer time to recover than patients who don’t show symptoms of COVID-19. Also, the rate of achieving recovery for patients who have other co-morbid cases in addition to COVID-19 was 83.73% lower as compared to patients with no co-morbid illness (AHR = 0.1627, 95% CI: 0.1396, 0.1897). That means the time needed to rich recovery for patients with the presence of co-morbid illness takes a longer time to recover as compared to patients with the absence of co-morbid illness.

The rate of achieving recovery for patients who have asthma was 49.45% lower as compared to patients who haven’t asthma (AHR = 0.5415, 95% CI: 0.3830, 0.7657). That means the time needed to attain recovery for patients who have asthma was longer. Also, the rate of achieving recovery from COVID-19 for patients who have the presence of respiratory disease was 76.99% times lower as compared to patients who haven’t a respiratory disease (AHR = 0.2301, 95% CI: 0.0792, 0.6607). That means the time needed to reach recovery for patients who have any respiratory disease was longer. The rate of achieving recovery for patients having oxygen saturation of above 90 was 3.237 times higher than for patients having less than or equal to 89 (AHR = 3.237, 95% CI: 2.161,4.848). Finally, for a unit increase in age, the rate of achieving recovery from COVID-19 for patients was decreased by 1.60% (AHR = 0.9840, 95% CI:0.971, 0.9973).

### Model diagnostics

Once the model is fitted, the next step is to verify the entire necessary model assumptions are valid in the selected model. To check these model assumptions, we often make use of standard types of residual plots to validate the assumptions behind the Cox PH model.

**Fig. 2:**
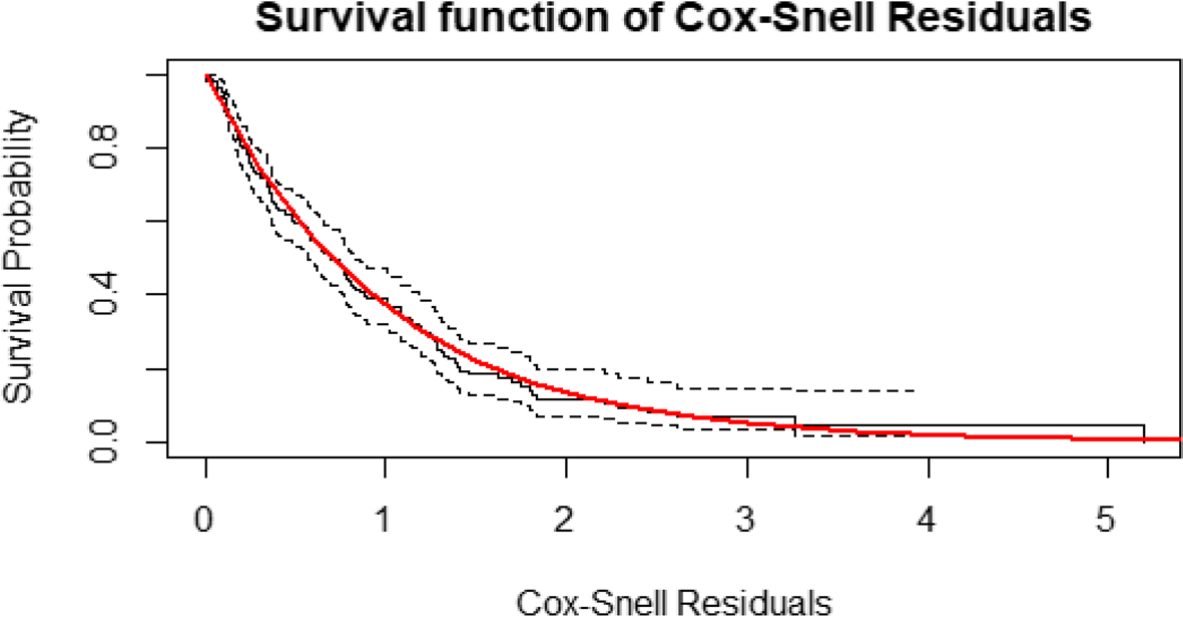
Cox-snell residual plots for time to recovery for COVID-19 patients

**Fig. 3:**
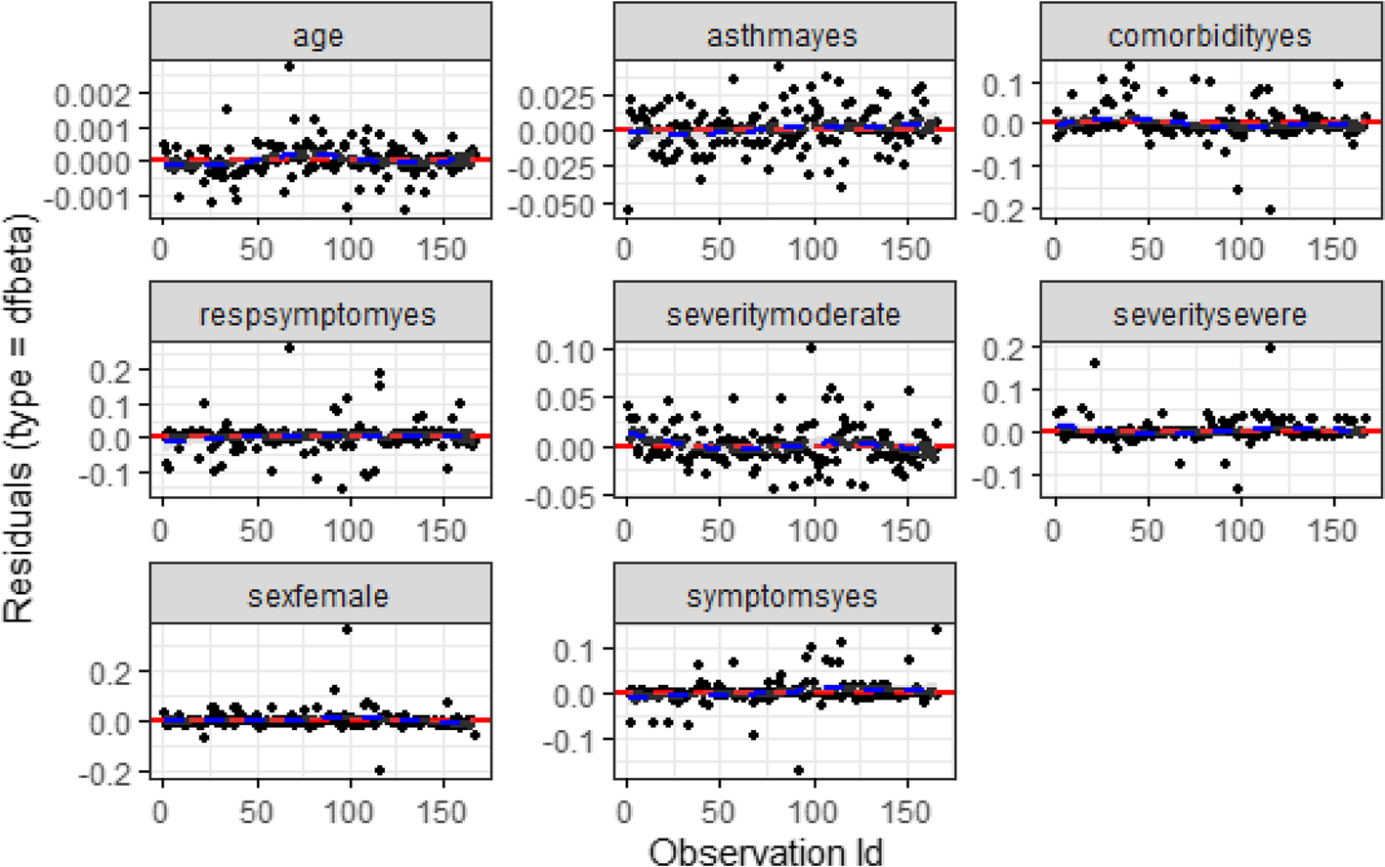
Residual plots for significant predictors

From (Fig. 2), the diagnostic based on Cox-Snell residuals with the 95% point-wise CI for the Kaplan–Meier estimate of the Cox-Snell residuals along the red line. The survival function of the unit exponential distribution indicates that the survival function of the standard exponential distribution lies within the 95% CI of the Kaplan–Meier estimate. This indicates the survival process model fits the data well. Also (Fig. 3) showed that the Dfbeta residual plots were randomly distributed and a loess-smoothed curve does not exhibit more departure from the horizontal line or the origin. All the above two residual plots indicated that the selected survival model (Cox PH model) fits the data well.

## Discussion

In this study, we assessed the determinant factors that prolonged or shortened the recovery times for COVID-19 patients who were admitted at Assosa COVID-19 treatment center. This study demonstrated that the median time of recovery from COVID-19 was 16 days. This is more or less similar to the result obtained from the studies [8, 28, 29]. This might be due to the treatment given to the patients being somewhat the same and also this might be due to similar characteristics of the study participants based on the factor variables. However, this result was contradicted to another study [27, 31]. This might be due to the immunity of study participants in their study being better. That means patients in their study might; have a better intake of supplements and fortified food, be free of stress, have better sleep–wake cycles of circadian rhythms, and have better hygiene before they entered the treatment center. These all have a positive effect on the immunity system. Furthermore, the observed difference might be due to sample size, patient characteristics difference, and study setting.

The Kaplan- Meier curve & log-rank test shows that female COVID-19 patients, patients having one or more symptoms at presentation, presenting with respiratory symptoms, patients having other co-morbid illness in addition to COVID-19 & patients having asthma seems to extend the time needed to achieve recovery. That means the recovery time for these patients was delayed or prolonged as compared to the reference categories.

Age was an important clinical variable of time to recovery implying that as the age of the patient increase the rate of attaining recovery decrease. This result was consistent with another study [7, 29, 31]. In their finding, the time to recovery decreases with increased age. This might be due to older aged individuals’ tendency in being with comorbidity, immunity curtailment and other underline condition that leads to a delayed recovery time from COVID-19.

In this study, we found a significant association between the sex of the study participants and the time to recover from COVID-19. This result was consistent with a study [19, 27]. In their finding, Female patients recover in a shorter time as compared to male patients. This result was also consistent with another study [36]. In their finding, females had a lower incidence of SARS-CoV-2 infection than males. This sex difference might be due to androgen hormones since men have higher plasma levels as compared to women, which also drive the transcription of TMPRSS2, the gene coding for the protease essential for SARS-CoV-2 cell entry following the biding of its spike protein to cell membrane ACE2 [37]. However, This study was contradicted by another study [38]. This study was also contradicted by another study about covid-19 situation in India [39]. In their finding, the recovery time of male and female patients was nearly similar. Patients who had related co-morbid illness prolonged recovery time. This result was consistent with another study [8, 29, 31, 32]. In their finding, the study participants without co-morbidities recovered more quickly than those having co-morbidity. This result was also supported by the study [13]. In their finding, patients having hypertension and intravenous immunoglobulin may delay the viral clearance in COVID-19 patients. This might be due to patients having related co-morbidity their immunity might be decreased because of that additional disease. Comorbidity might not only have a single effect but also have a combined effect (comorbidity with increased age) on suppression of immunity. This is because of the presence of a common relationship between comorbidity and age of individuals. However, both affect delaying recovery time from COVID-19.

However, this result was contradicted with another study [40]. In their finding, asthma in hospitalized COVID-19 patients was associated with a lower risk of mortality and no increase in disease severity in hospitalized COVID-19 patients. This might be due to hospitalization or treatment difference, if the patients having asthma are treated in a good manner they may recover quickly without disease severity.

Patients having one or more symptoms at presentation delayed the recovery time from COVID-19. This result was consistent with another study [28]. In their finding, symptomatic patients are more likely to be infectious because of the prolonged viral shedding in addition to the presence of a more concentrated virus in the upper respiratory tract that enhances the transmission. This result was more or less consistent with another study [17]. In their finding, patients having higher symptom duration delayed their recovery time. Also, this result supported the study [41]. Their finding suggests that patients having symptoms who recovered from COVID-19 disease may still experience COVID-19 symptoms, particularly fatigue and headaches. This might be depending on the severity of the disease; patients having severe COVID-19 may experience COVID-19 symptoms even if they have recovered from the disease.

Patients having severe COVID-19 cases take a longer time to recover. This result was consistent with another study [37]. In their finding, the time from chemosensory loss to recovery for the patients who recover was associated with the severity of impairment. That is, less severe hyposmia tends to recover quickly.

### Strength and limitation of the study

This study was done by a well-defined statistical model and this should give a more appropriate result. Since there were scarce of studies done in Western Ethiopia, this finding was used as input for other studies who wanted to do research in this area or anywhere. Our findings were also subject to some limitations. There could be a chance that the duration of mild symptoms might be overlooked by individuals and not reported, as a result, our sample size was smaller than expected. The study could not identify other known clinical predictors of COVID-19 due to the small number of observations of severe COVID-19 cases treated at the treatment center. There were additional important variables that couldn’t be recorded in the patient’s medical records that might have a significant influence on the duration of recovery from COVID-19.

## Conclusion

In general, this study found the factors that prolonged or shortened the recovery time for COVID-19 patients. The study concludes that severe COVID-19 patients, male patients, patients having other related diseases, older age, and patients having symptoms as poor prognostic factors of COVID-19 disease and also prolonged recovery time. Therefore, health providers in treatment centers should give strict follow-up and priority to older patients, severe COVID-19 patients, and patients having another co-morbid illness by focusing on respiratory difficulties and underlying pre-existing medical conditions to manage the disease severity and recover quickly.

## Data Availability

The data used for the current investigation is available in the hands of the corresponding author and will be submitted upon request.

## Abbreviations

Respsymptom: Respiratory symptom
Respdisease: Respiratory disease
SD: Standard Deviation
Chisq: Chi-Square
SE: Standard Error
WHO: World Health Organization
APACHE-II: Acute Physiological and Chronic Health Evaluation II
BMI: Body Mass Index
Cox-PH: Cox Proportional Hazard
BGRS: Benishangul Gumez Reginal State
ARDS: Acute Respiratory Distress Syndrome
CI: Confidence Interval
AHR: Adjusted hazard ratio
KM: Kaplan- Meier
